# Low-energy shock wave therapy ameliorates ischemic-induced overactive bladder in a rat model

**DOI:** 10.1038/s41598-022-26292-x

**Published:** 2022-12-19

**Authors:** Shingo Kimura, Naoki Kawamorita, Yoku Kikuchi, Tomohiko Shindo, Yuichi Ishizuka, Yohei Satake, Takuma Sato, Hideaki Izumi, Shinichi Yamashita, Satoshi Yasuda, Hiroaki Shimokawa, Akihiro Ito

**Affiliations:** 1 Department of Urology, Tohoku University Graduate School of Medicine, 1-1 Seiryo-machi, Aoba-ku, Sendai, 980-8574 Japan; 2 Department of Cardiovascular Medicine, Tohoku University Graduate School of Medicine, Sendai, Japan; 3 Graduate School, International University of Health and Welfare, Narita, Japan

**Keywords:** Urological manifestations, Ageing, Membrane biophysics

## Abstract

This study was to evaluate whether Low-energy shock wave therapy (LESW) improves ischemic-induced overactive bladder in rats and investigate its therapeutic mechanisms. Sixteen-week-old male Sprague–Dawley rats were divided into three groups: arterial injury (AI), AI with LESW (AI-SW), and control groups. LESW was irradiated in AI-SW during 20–23 weeks of age. At 24 weeks of age, conscious cystometry was performed (each n = 8). The voiding interval was shortened in AI (mean ± SEM: 5.1 ± 0.8 min) than in control (17.3 ± 3.0 min), whereas significant improvements were observed in AI-SW (14.9 ± 3.3 min). The bladder blood flow was significantly increased in AI-SW than in AI. Microarray analysis revealed higher gene expression of soluble guanylate cyclase (sGC) α1 and β1 in the bladder of AI-SW compared to AI. Protein expression of sGCα1 and sGCβ1 was higher in AI-SW and control groups than in AI. Cyclic guanosine monophosphate (cGMP) was elevated in AI-SW. As an early genetic response, vascular endothelial growth factor and CD31 were highly expressed 24 h after the first LESW. Suburothelial thinning observed in AI was restored in AI-SW. Activation of sGC-cGMP may play a therapeutic role of LESW in the functional recovery of the bladder.

## Introduction

Lower urinary tract symptoms (LUTS) are influenced by multiple factors, including bladder outlet obstructions, neurological diseases, psychosocial conditions, and various aging-related medical diseases^1–3^. Of particular note, chronic pelvic ischemia related to metabolic syndrome is considered to have a preeminent role in causing a storage symptom syndrome, “overactive bladder (OAB),” in the aging population^4^. According to studies on animal models of chronic bladder ischemia, increased oxidative stress and proinflammatory cytokines upregulate various stimulatory molecules in the urothelium and the suburothelium^5–7^. These processes result in stimulating afferent nerve activity and frequent urinary voiding^4^.

OAB has been treated with medications including anti-muscarinics, beta-3 agonist and phosphodiesterase (PDE) 5 inhibitors. Especially, PDE 5 inhibitors are effective for ischemia-related symptoms for animals and humans^8,9^. Invasive treatment of botulinum toxin injection or sacral neuromodulation would be selected for severe cases; however, there is still a strong demand of non-invasive treatment for refractory OAB.

In recent years, low-energy shock wave therapy (LESW) has become known to be effective in inducing various biological changes including angiogenesis, anti-inflammation, nerve regeneration, cell proliferation, and alteration of membrane permeability^10^. In fact, LESW is being used clinically in cardiovascular diseases^11,12^, musculoskeletal disorders^13^ and erectile dysfunction^14^. A clinical pilot study for OAB has recently been launched and its outcome has been positive to date^15^.

We hypothesized the therapeutic effect of LESW on OAB induced by chronic bladder ischemia. Using a rat model of ischemic-induced OAB, with a combination of arterial injury and a high-cholesterol diet^5^, we evaluated whether LESW improved urinary frequency and investigated its therapeutic mechanisms.

## Results

### Cystometry

**Figure 1:**
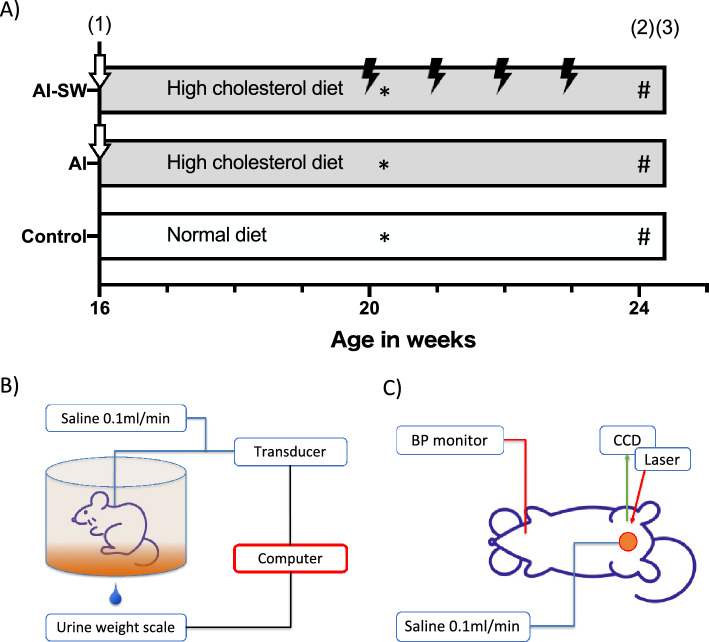
(**A**) Experimental design. (1) AI-SW and AI models underwent arterial injury at 16 weeks of age (arrows) and received a high cholesterol diet. LESW was irradiated once a week during 20–23 week (thunder mark). (2) Catheter was implanted into the bladder at 24 weeks of age. (3) Three days later, cystometry and blood flow measurement were performed. For molecular evaluations, animals without catheter implantation were prepared. The bladder was harvested 24 h after LESW (20 weeks of age) for the early phase (*), and one week after the fourth LESW (24 weeks of age) for the late phase (#).**(B**) A Conscious cystometry was performed in a free-moving metabolic cage. (**C**) A rat was anesthetized and the anterior wall of the bladder was exposed to measure the blood flow using laser speckle imaging. Arterial blood pressure was simultaneously monitored from the left carotid artery.

**Figure 2:**
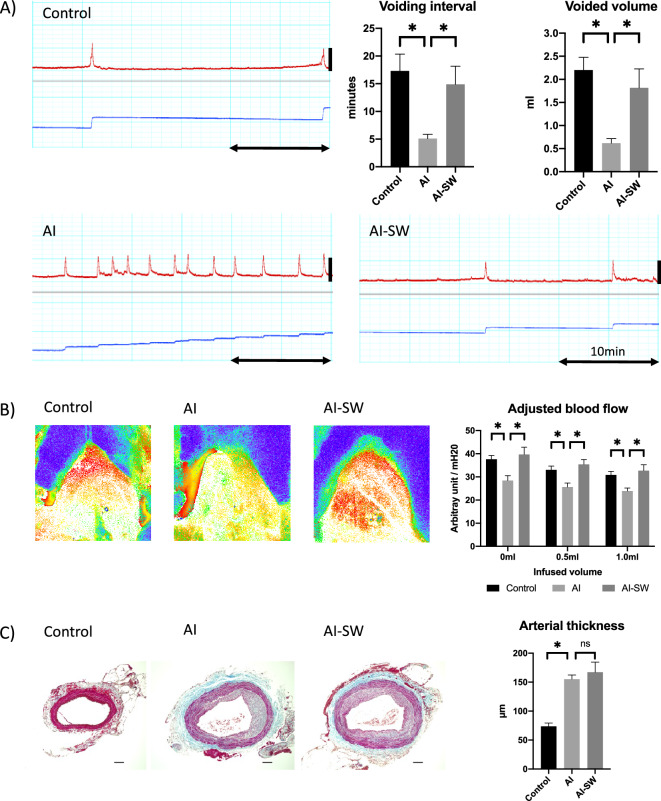
(**A**) Cystometry. Urinary frequency was significantly increased in the AI group than the AI-SW and control groups. Red lines show intravesical pressure and blue lines are voided urine volume. The double horizontal arrow represents the duration of 10 min. The vertical bars indicate 0–50 cmH20. (**B**) Bladder blood flow measurement. Blood flow was measured in the anterior bladder walls and adjusted by arterial blood pressure. The adjusted blood flow was significantly greater in the control and AI-SW groups than in the AI group at the three points of saline infusion (0, 0.5, and 1.0 ml).(**C**) Masson’s trichrome staining of the common iliac arteries. The arterial wall in the AI and AI-SW groups were significantly thicker than in the control group. There was no statistical difference between the AI and AI-SW groups. Bars indicate 100 μm. *, P < 0.05. ns, not significant. All data are presented as mean ± SEM.

Sixteen-week-old male Sprague–Dawley rats were divided into three groups (each n = 8): arterial injury (AI), AI with LESW (AI-SW), and control groups (Fig. 1A). LESW was irradiated in AI-SW during 20–23 weeks of age. At 24 weeks of age, conscious cystometry was performed (Fig. 1B). There was no statistical difference in body weight among rats in the three groups at the time of AI procedure at 16 weeks of age or at the time of cystometry at 24 weeks of age. In conscious cystometry, voided volume, bladder capacity and voiding interval decreased significantly in the AI group than in the control group, while those parameters improved significantly in the AI-SW group (Fig. 2A, Table 1). Post-void residual (PVR) volume was less than 0.1 ml in all groups. There was no significant difference in baseline pressure, threshold pressure, or maximum pressure. Bladder compliance decreased in the AI group without statistical significance.

**Table 1:** Body weight and cystometric parameters.

	Control	AI	AI-SW
Number of rats	8	8	8
Body weight at 16 weeks of age (g)	551 ± 7.3	527 ± 8.73	531 ± 9.76
Body weight at 24 weeks of age (g)	639 ± 7.7	613 ± 14.3	641 ± 15.9
Total voided volume (ml)	12.5 ± 0.76	11.7 ± 0.62	12.2 ± 0.91
Voiding frequency (times)/90 min	6.25 ± 0.86	19.9 ± 2.55*	8.75 ± 1.62#
Mean voided volume (ml)	2.23 ± 0.77	0.65 ± 0.22*	1.84 ± 1.16#
Bladder capacity (ml)	1.73 ± 0.30	0.51 ± 0.08*	1.49 ± 0.33#
Voiding interval (min)	17.3 ± 3.04	5.1 ± 0.76*	14.9 ± 3.26#
Post-void residual (ml)	< 0.1	< 0.1	< 0.1
Baseline pressure (cmH2O)	6.0 ± 0.7	10.3 ± 1.4	8.0 ± 1.5
Threshold pressure (cmH2O)	14.4 ± 0.84	17.8 ± 1.13	15.1 ± 2.55
Maximum pressure (cmH2O)	69.6 ± 2.72	70.3 ± 3.67	66.6 ± 6.27
Bladder Compliance (ml/cmH2O)	0.25 ± 0.088	0.068 ± 0.0054	0.26 ± 0.079

Threshold pressure, pressure at which voiding was initiated; bladder capacity, infusion volume between voids; bladder compliance, bladder capacity divided by the difference between threshold and baseline pressure; *, *P* < 0.05 versus control; #, *P* < 0.05 versus AI. None of the parameters were significant between the control and AI-SW groups. Data are presented mean ± SEM. Post-void residual volumes were less than 0.1 ml in all animals.

### Bladder blood flow

After conscious cystometry, the bladder was exposed to measure blood flow under anesthesia (Fig. 1C). The bladder blood flow adjusted by arterial blood pressure decreased significantly in the AI group compared to the control group, while the blood flow of the AI-SW group increased at each of the three points of saline infusion: 0, 0.5 and 1.0 ml (Fig. 2B).

### Thickness of arterial wall

The common iliac arteries were harvested from animals sacrificed after cystometry and blood flow measurement. Neo-intima formation was observed in the arterial wall of the AI and AI-SW groups. The thickness of the arterial wall measured in four directions was significantly greater in the AI and AI-SW groups than in the control group (Fig. 2C).

### Microarray

**Figure 3:**
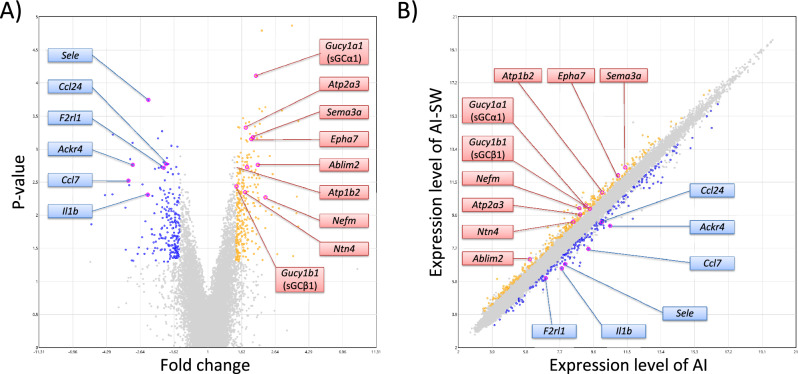
Results of microarray analysis. There were 294 up-regulated genes and 212 down-regulated genes in the AI-SW group against the AI group. (**A**) Volcano plot. (**B**) Scatter plot. Genes with fold change > 1.5 and *P*-value < 0.05 were plotted with yellow dots and < 1.5 with blue dots. The putative candidates were marked as pink dots with annotation.

Animals without catheter implantation were prepared for molecular evaluations and the bladder was harvested. Microarray analysis was performed for isolated RNA samples from AI-SW and AI groups (n = 4 each) at 24 weeks of age. Of the total 23,188 genes, microarray analysis identified 294 up-regulated genes and 212 down-regulated genes in the AI-SW group (n = 4) against the AI group (N = 4) with fold change >|1.5| and P-value < 0.05 (Fig. 3). Of particular note, *Gucy1a1* coding soluble guanylate cyclase α1 (sGCα1) and *Gucy1b1* coding soluble guanylate cyclase β1 (sGCβ1) were significantly up-regulated in the AI-SW group: 2.00 folds with p-value of 0.000077 and 1.51 folds with p-value of 0.0037, respectively. We also observed up-regulation of several genes related to axon guidance and neurogenesis (*Ablim2, Epha7, Sema3a, Nefm and Ntn4*) and genes coding ATPase (*Atp1b2 and Atp2a3*) in the AI-SW group, while some inflammation-related genes (*Sele, Il1b, Ccl7, Ccl24, and F2rl1*) were downregulated.

### Real-time PCR

**Figure 4:**
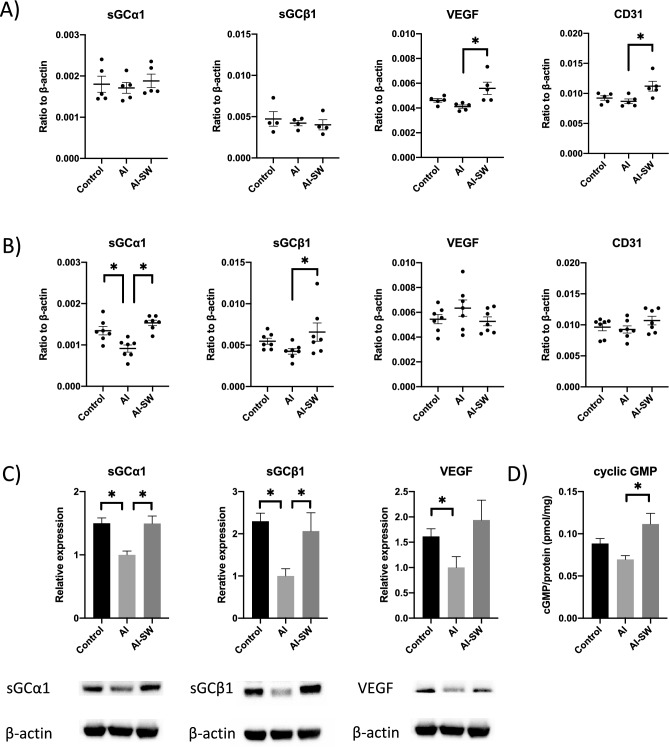
(**A**) Gene expression at the early phase (24 h after the first LESW) by real-time PCR (ratio to β-actin). Significant upregulations of VEGF and CD31 were observed in the AI-SW group than in the AI group in the early phase. There was no significant difference among three groups in sGCα1 or sGCβ1. (**B**) Gene expression at the late phase (24 weeks of age) by real-time PCR (ratio to β-actin). sGCα1 expressions were significantly higher in the control and AI-SW groups than in the AI group in the late phase. There was also higher expression of sGCβ1 in the AI-SW than in the AI group. VEGF expression was higher in the AI group without statistical significance. (**C**) Protein expression at the late phase by Western blotting (relative expression /β-actin). The expressions of sGCα1 and sGCβ1 were significantly higher in the control and AI-SW groups than in the AI group. VEGF was highly expressed in the control than in the AI group. (**D**) Cyclic GMP assay. The amount of cGMP (/total protein) was significantly greater in the AI-SW group than in the AI group. *, *P* < 0.05. All data are presented as mean ± SEM in the graphs.

Real-time PCR was performed on five 20-week-old rats from each group (24 h after the first LESW: the early phase) and seven 24-week-old rats (one week after the fourth LESW: the late phase) from each group. There was a significantly higher expression of VEGF and CD31 in the AI-SW group for 20-week-old rats than in the AI group (Fig. 4A).For the 24-week-old rats, expression of sGCα1 was significantly higher in the control and AI-SW groups than in the AI group (Fig. 4B). sGCβ1 expression was also higher in the AI-SW group than in the AI group. There was no significant change in VEGF or CD31 among rats in the three groups at 24 weeks of age.

### Western blotting

Western blotting was performed on the extracted protein from the bladder tissues of five 24-week-old rats (Fig. 4C). Expressions of sGCα1 and sGCβ1 were significantly higher in the control and the AI-SW groups than in the AI group. VEGF was decreased in the AI compared to the control group.

### Cyclic guanosine monophosphate (cGMP) assay

cGMP assay was performed for the bladder tissue of 24-week-old rats (n = 7 each) (Fig. 4D). There was a significantly larger amount of cGMP in the AI-SW group than in the AI group.

### Histological examinations of the bladder

**Figure 5:**
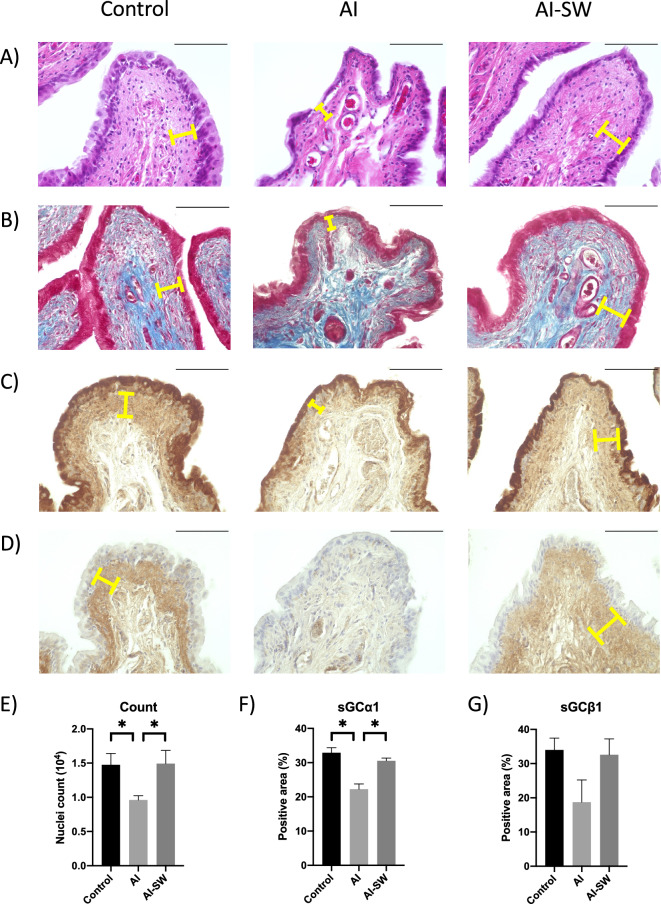
(**A**) Hematoxylin and Eosin (HE) staining and (**B**) Masson’s trichrome staining of the bladder. The suburothelial layer (yellow bars) is thinned in the AI model, while the thinning restored in AI-SW model. (**C**, **D**) Immunohistochemistry (IHC) of sGCα1 (**C**, G4280, Sigma-Aldrich), and sGCβ1 (**D**, NBP1-89,784, Novus Biologicals). Suburothelial layer is weakly stained in the AI model while a strong deposition can be seen in the AI-SW and control model. Black bars indicate 100 µm. (**E**) Quantitative analysis of HE staining. Number of hematoxylin-stained nuclei in lamina propria was significantly decreased in the AI group compared to the AI-SW and control groups. (**F**, **G**) Quantitative analyses of IHC. The expression of sGCα1 was significantly decreased in the AI group.

On Hematoxylin and Eosin (HE) staining and Masson’s trichrome staining demonstrated thinning of the suburothelial layer in the AI model, while the thinning was restored in the AI-SW model (Fig. 5A, B). In quantitative analysis, number of hematoxylin-stained nuclei in lamina propria was significantly decreased in the AI groups compared to the AI-SW and control groups (Fig. 5E). In immunohistochemistry (IHC), sGCα1 and sGCβ1 were deposited in the suburothelial layer of the AI-SW and control groups (Fig. 5C, D) Quantitative analyses of IHC demonstrate statistical difference in sGCα1 (Fig. 5 F, G).

## Discussion

Our study demonstrated that LESW improved urinary frequency in a rat model of chronic bladder ischemia along with increased blood flow of the bladder. Following the results of microarray analysis, the subsequent examinations showed higher expressions of sGC and cGMP after LESW.

sGC is a heterodimeric complex consisting of α-subunit and hem-binding β-subunit, which converts guanosine triphosphate (GTP) to cGMP as an intracellular receptor of nitric oxide (NO)^16^. Then, cGMP induces relaxation of vascular smooth muscle via activation of protein kinase G (PKG), which increases blood perfusion^17^. In the lower urinary tract, cGMP plays some critical roles, including relaxation of smooth muscle of the urethra and the detrusor as a PDE5 inhibitor has been a therapeutic option for LUTS^18,19^. Moreover, activation of sGC-cGMP is considered to inhibit afferent nerve stimulation in the bladder. There is some evidence that the activation of eNOS and NO inhibits afferent nerves (Aδ and C fibers) in the bladder^20,21^, and PDE 5 inhibitor suppresses neuropeptide release at afferent nerve terminals^22,23^. Meanwhile, oxidative stress caused by ischemia degrades sGC as a reduced hem (Fe^2+^) shifts to an oxidized form (Fe^3+^)^16^. Leiria et al.^24^demonstrated decreased protein expression of sGC in the bladder tissue in an obese mouse model, whereas a NO-independent sGC activator agent restored sGC expression and showed improvement in cystometric parameters.

Even though sGC and cGMP could be involved in therapeutic mechanisms of ischemic-induced OAB, little has been investigated about the activation of sGC-cGMP with LESW. In contrast, many studies of LESW demonstrated functional recovery of pathophysiology in animal models along with angiogenesis involved with VEGF^25–27^ and CD31^28^ and activation of eNOS^28^. In-vitro studies revealed that LESW upregulates VEGF and enhances NO production via activation of eNOS in human umbilical vein endothelial cells (HUVECs)^25,29^. Hatanaka et al.^30^demonstrated that shock wave therapy upregulates caveolin-1 and β1-integrin, which opens up considerations to its possibility to work as mechanosensors in caveolae on cell membranes. They also revealed enhanced phosphorylation of downstream mediators including Akt, Erk1/2, and FAK. This signaling pathway is considered to promote VEGF and eNOS expressions^30^. Despite lacking definitive evidence of angiogenesis other than the early genetic response of VEGF and CD31 after LESW, the angiogenesis could increase the blood perfusion, then, increased blood perfusion could prevent ischemic-induced degradation of sGC. Activation of eNOS, if it occurs, could also upregulate expression of sGC and cGMP. In the AI-SW model, activated cGMP may also increase peripheral perfusion with vessel dilation creating a virtuous cycle, while the AI model fails to promote cGMP due to a shortage of sGC under chronic ischemia.

It is also notable that protein expression of VEGF was decreased in the AI group in the late phase. Yang et al. demonstrated that gene expression of VEGF increased significantly in a rabbit model of chronic bladder ischemia while protein expression did not^31^. They discuss that the gene upregulation could be a defensive response to ischemia but fails to activate the downstream regulators to produce the VEGF protein.

In the histological aspect, the suburothelial layer (upper lamina propria) contains the dense capillary plexus, interstitial cells, and afferent nerve endings, while a deeper layer of lamina propria consists mainly of the extracellular matrix^32–34^. Even though our results are not sufficient to explain the evidence of angiogenesis, a change in the number of nuclei with LESW may indicate structural restoration including capillary plexus. Additionally, despite the non-specific distribution, the expression of sGC was notable in the suburothelium in the AI-SW and control models, while the expression was weak in the thinned suburothelium in the AI model. While there has been a little evidence of IHC study of sGC-cGMP in the bladder, Gillespie et al. reported that NO-dependent cGMP synthesis was observed primarily in the suburothelium just below the basal cells of the urothelium^35^. Our results could imply some involvement of sGC-cGMP in the suburothelium in the pathophysiology of chronic ischemia and also the therapeutic mechanism of LESW.

There are some limitations that must be considered in this study. First, we have yet to obtain definitive evidence of angiogenesis after LESW other than higher gene expression of VEGF and CD31. Further investigations are required to demonstrate angiogenesis. Second, upstream mediators, time-dependent change, and interaction of sGC and VEGF have yet to be investigated. sGC was possibly upregulated by the upstream mediators including eNOS and Akt derived from mechano-transduction^30^. Additional checkpoints throughout the early-late phases, other than those limited number of genes examined at the early phase, will be needed to follow the time-dependent change and the interaction of sGC, eNOS, and VEGF. Third, the only factors that improved the cystometric parameters after LESW may not be limited to activation of sGC-cGMP or angiogenesis. Wang et al. reported that voiding dysfunction in a diabetic rat improved as neuronal integrity and innervation recovered after shock wave treatment^36^. Several inflammatory mediators decreased with improved cystometric parameters after shock wave treatment in a mouse model of uroplakin 3A-induced cystitis^37^ and a rat model of cyclophosphamide-induced cystitis^38^. As the microarray analysis indicated, LESW can potentially involve neurogenesis, anti-inflammation, and other processes. Future works are needed for further understanding of the therapeutic mechanisms of LESW in ischemia-induced OAB.

In conclusion, LESW improved the capacity and blood flow of the bladder and brought an upregulation of cGMP in a model of chronic bladder ischemia. Activation of the sGC-cGMP pathway may play a crucial role in the functional recovery of the bladder. This study indicates that LESW can be a novel therapeutic option for OAB.

## Materials and methods

### Ethics statement

The protocol of this study was approved by the Animal Experiment Committee at Tohoku University (Approved number: 2019MdA-226). All experiments were performed in following Regulations for Animal Experiments and Related Activities at Tohoku University and the Animal Research: Reporting of In Vivo Experiments Guidelines. All efforts were made to minimize the number and suffering of the animals used.

### Animal preparations

Male Sprague–Dawley rats weighing 500-570 g at 16 weeks of age were randomly divided into three groups: arterial injury (AI), AI-SW, and control groups (Fig. 5A). The AI and AI-SW groups underwent arterial injury at 16 weeks of age and received a 2% cholesterol diet for eight weeks. In the AI-SW group, LESW was conducted once a week over four weeks from 20 to 23 weeks of age. A regular diet, which included 0.09% cholesterol, was administered to rats in the control group.

### Arterial injury

Rats were anesthetized with 3% isoflurane, and a 2-Fr Fogarty arterial embolectomy catheter (E-060-2F; Edwards Lifesciences LLC, Irvine, CA) was inserted into the common iliac artery through an incision in the femoral artery. A balloon was inflated with air and subsequently withdrawn from the common iliac artery to the femoral artery,　as previously reported^39^. This withdrawal maneuver was repeated 20 times on each side. Then, the arteries were ligated, and the skin was sutured.

### Shock wave therapy

Shock wave generator DUOLITH SD1 (Storz Medical, Tägerwilen, Switzerland) was used to give 1800 shots with an intensity of 0.25 mJ/mm^2^ (total energy flux density) and frequency of 3 Hz. The intensity is equivalent to 0.1 mJ/mm^2^ of positive energy flux density and is considered the most effective for angiogenesis^25,26,28^. The focused handpiece was manually placed on the lower abdominal wall and animals were anesthetized during the procedure. According to the manufacturer's protocol, the focal point of the shock wave was within 10 mm of depth from the probe’s tip.

### Voiding cystometry

At 24 weeks of age, the PE-50 polyethylene tubing (BD, Franklin Lakes, NJ) was implanted into the bladder for eight rats from each group. The PE-50 tubing was then tunneled subcutaneously and exited through the skin from the back of the rat. Three days after this procedure, cystometry was performed in conscious, freely moving rats in a metabolic cage (Fig. 1B). Saline was infused at a rate of 0.1 ml/min with an infusion pump. Bladder pressure and urine volumes were monitored by PowerLab (Ad Instruments, Colorado Springs, CO). After a stable voiding pattern was obtained, voided volumes and intravesical pressures were measured for more than 90 min.

### Measurement of bladder blood flow

After conscious cystometry, rats were placed under anesthesia and the bladder was exposed. After the drainage of urine, blood flow of the anterior bladder wall was repeatedly measured each at 0, 0.5 and 1.0 ml of saline infused at a rate of 0.1 ml/min using a laser speckle imaging system (OMEGAZONE; Omegawave, Tokyo, Japan) (Fig. 1C) in a room shielded from natural light exposure. The measured value was adjusted to the ratio to arterial blood pressure, which was simultaneously monitored by cannulating the left carotid artery. When the measurements of the blood flow were complete, rats were euthanized with potassium chloride.

### Total RNA extraction

Bladders were harvested from animals without undergoing cystometry and stored at − 80 °C. Total RNA was isolated from all groups using the RNeasy Mini Kit (QIAGEN, Hilden, Germany).

### Microarray analysis

Microarray analysis was performed for isolated RNA samples from AI-SW and AI groups (n = 4 each) at 24 weeks of age with Affymetrix Clariom™ S Assay, rat (Thermo Fisher Scientific, Waltham, MA). The data files obtained were analyzed using Affymetrix Transcriptome Analysis Console software.

### Real-time polymerase chain reaction (PCR)

Real-time PCR was performed with a Dice Real Time System Thermal Cycler (TAKARA, Shiga, Japan) using SYBR Premix Ex Taq™ II (TAKARA). The protocol consisted of 40 replication cycles. Gene expressions were quantified considering β-actin as a housekeeping gene. The primer sequences were obtained from the following accession numbers: sGCα1 (*Gucy1a1*), NM_012769.2; sGCβ1 (*Gucy1b1*), NM_012769.2; VEGF (*vegfa*), NM_031836.3; CD31 (*Pecam1*), NM_031591.2; and β-actin (*Actb*), NM_031144.2. ﻿The data of accession numbers in this study are available in the NCBI depository (https://www.ncbi.nlm.nih.gov/nuccore/).

### Protein extraction

Total protein was extracted from the bladder of rats at 24 weeks of age without undergoing cystometry by Tissue Protein Extraction Reagent (T-PER, Thermo Fisher Scientific) with Halt™ Protease Inhibitor and Phosphatase Inhibitor Cocktail (Thermo Fisher Scientific). The protein concentration was measured by Pierce™ BCA Protein Assay Kit (Thermo Fisher Scientific).

### Western blotting

Protein samples were denatured with 4X Laemmli Sample Buffer (Bio-Rad Laboratories, Inc., Hercules, CA) and 2-mercaptoethanol and loaded onto 10% SDS-PAGE gel electrophoresis. The transferred PVDF membrane was cut into three parts prior to hybridization with antibodies. Then, overnight incubation was performed with primary antibodies: anti-sGCα1 subunit (1:4000, G4280, Sigma-Aldrich, St-Louis, MO), anti-sGCβ1 subunit (1:2000, NBP1-89,784, Novus Biologicals, Littleton, CO), anti-β actin (1:10,000, MAB8929, R&D systems, Minneapolis, MN), and anti-VEGF (1:200, sc-7269, Santa Cruz Biotechnology, Dallas, Texas). HRP-conjugated secondary antibodies were anti-mouse (1:4000, G-21040, Invitrogen, Carlsbad, CA) and anti-rabbit (1:2000, G-21234, Invitrogen). Protein bands were visualized with the ChemiDoc Touch Imaging System (Bio-Rad) and quantified using Image Lab software (Bio-Rad).

### Cyclic guanosine monophosphate (cGMP) assay

The extracted protein of the bladder was processed for cGMP measurement. We measured cGMP using the Cyclic GMP EIA Kit (Cayman Chemical, Ann Arbor, MI) in accordance to the manufacturer’s protocol. All samples were acetylated immediately before the assay. Assay was performed in duplicate and the ratio of cGMP concentration to the total protein concentration was observed for group comparison.

### Histological examinations

The bladder and the common iliac artery were harvested from rats at 24 weeks of age and fixed in fresh 4% paraformaldehyde (pH 7.4). Modified Masson's trichrome staining was performed using Trichrome Stain Kit (Scy Tek Laboratories, Logan, UT). IHC staining was performed with same antibodies used in Western blotting. Antigen retrieval was performed in citrate buffer solution (10 mM, pH 7.0) at 70ºC for 60 min. Background activity was blocked with Protein Block (Dako, Glostrup, Denmark) at room temperature for 1 h. Reaction products were visualized by EnVision + System-HRP Labeled Polymer (Dako) and the Liquid DAB + Substrate Chromogen System (Dako). Number of nuclei was counted from Hematoxylin-stained nuclei of lamina propria (urothelium and muscle layer were subtracted) of the whole circumference of the bladder using ImageJ software (https://imagej.nih.gov/ij/). IHC staining was quantified by measuring the stain-positive area ratio for all layers of the bladder from each slide using ImageJ software.

### Statistical analysis

All values are presented as means ± standard error of the mean. We used JMP Pro version 15.2.0 for statistical analysis. One-way analysis of variance followed by the Tukey test was applied to all groups with p-value < 0.05 considered significant.

## Data Availability

The datasets used and analysed during the current study available from the corresponding author on reasonable request.
