# Author Correction: Low-energy shock wave therapy ameliorates ischemic-induced overactive bladder in a rat model

**DOI:** 10.1038/s41598-023-39073-x

**Published:** 2023-07-25

**Authors:** Shingo Kimura, Naoki Kawamorita, Yoku Kikuchi, Tomohiko Shindo, Yuichi Ishizuka, Yohei Satake, Takuma Sato, Hideaki Izumi, Shinichi Yamashita, Satoshi Yasuda, Hiroaki Shimokawa, Akihiro Ito

**Affiliations:** 1 Department of Urology, Tohoku University Graduate School of Medicine, 1-1 Seiryo-machi, Aoba-ku, Sendai, 980-8574 Japan; 2 Department of Cardiovascular Medicine, Tohoku University Graduate School of Medicine, Sendai, Japan; 3 Graduate School, International University of Health and Welfare, Narita, Japan

Correction to: *Scientific Reports* 10.1038/s41598-022-26292-x, published online 19 December 2022

The original version of this Article contained an error in the spelling of the author Yohei Satake which was incorrectly given as Yoichi Satake.

The original Article has been corrected.

